# Dr. Asbel Smith

**Published:** 1886-03-20

**Authors:** 


DR. ASBEL SMITH, an old and eminent member of the profession, a resident of Houston, Tex., died on January 21, 1886. He was the Surgeon-General of the Texas Republic, and also appointed Minister to France before its admission into the Union. He was prominent as a surgeon in the Mexican war ; had command of a regiment in the Confederate service ; was several times elected to the legislature of Texas, and in 1881 was made president of the Texas State Medical Association.

